# Complications and Mortality of Single Versus Dual Chamber Implantable Cardioverter Defibrillators

**Published:** 2006-04-01

**Authors:** Ataallah Bagherzadeh, Zahra Emkanjoo, Majid Haghjoo, Maryam Moshkani Farahani, Abolfath Alizadeh, Mohammad Ali Sadr-Ameli

**Affiliations:** Department of Pacemaker and Electrophysiology, Rajaie Cardiovascular Medical and Research Center, Iran University of Medical Sciences, Mellat Park, Vali-e-Asr Avenue, Tehran, Iran

**Keywords:** implantable cardioverter-defibrillator, complication, mortality

## Abstract

**Background:**

The implantable cardioverter defibrillators (ICDs) are increasingly being used as a treatment modality for life threatening tachyarrhythmia. The purpose of this study was to compare the frequency of complications and mortality between single-chamber and dual-chamber ICD implantation in Shahid Rajaie cardiovascular center.

**Methods and results:**

Between January 2000 and December 2004, 234 patients received ICD by a percutaneous transvenous approach and were followed for 33 ± 23 months. The cumulative incidence of complications was 9.4% over the follow-up period. There was no significant difference in overall complication rate between single chamber (VR) and dual chamber (DR) ICD groups in the follow-up period (P= 0.11). The risk of complications did not have any statistically significant difference in secondary versus primary prevention groups (P=0.06). The complications were not associated with the severity of left ventricular systolic dysfunction (P=0.16).The frequency of lead-related complications was higher in dual chamber ICDs in comparison with single chamber ICDs (P=0.02). There was no significant difference in mortality between different sex groups (P=0.37), different indications for ICD implantation (P=0.43) or between VR and DR ICD groups (P= 0.55). Predictors of mortality were NYHA class III or more (P<0.001), age >65 years (P=0.011) and LVEF<30% (P<0.001). The mortality in patients with CAD and DCM were significantly higher than those with other structural heart diseases (P=0.001).

**Conclusion:**

Close monitoring of patients during the first 2 month after ICD implantation is recommended because the majority of complications occur early after the procedure.

## Introduction

The implantable cardioverter defibrillators (ICDs) are increasingly being used as a treatment modality for high risk survivors of life-threatening tachyarrhythmia [1,2]. Major technological advances like the introduction of transvenous defibrillation leads and "active can" concept have simplified the implantation of ICDs and have reduced implantation-related complications but still there are considerable rate of complications in most of the electrophysiologic centers [3,4].

The purpose of the current study was to identify and characterize prospectively the frequency of lead and implant-related complications and also mortality in patients with single-chamber versus dual-chamber ICD implantation.

## Methods

### Patients

Between January, 2000 and December, 2004, 234 patients received ICDs in our center. All of them included in the study and were followed for 33 ±23 months. The primary end point of the study was actuarial survival. The secondary end point was nonfatal complications, including inappropriate ICD therapy, lead-related and implant-related complications. The study was approved by local Ethics Committee and written informed consent was obtained from all the patients.

### Implanted ICDs and programming

ICD system manufacturers included Medtronic and St Jude systems in 160 and 75 patients respectively. Atrial leads included 1688 T or 1642T for St Jude and 5076 for Medtronic systems. Ventricular leads included 1570,1572,1571,1580 and 1581 for St Jude and 6944, 6947 and 6948 for Medtronic systems. All of the ventricular leads were bipolar. In implanted devices all the detection and discrimination criteria were activated with the nominal values. In all the devices we defined ventricular fibrillation zone (300ms) plus one VT zone (400 ms). If the patient had an episode of spontaneous or induced sustained monomorphic VT slower than 370 ms we extended the VT zone to VT cycle length plus 40ms. In the VT detection zone the first therapy was three antitachycardia burst pacing. We used the nominal values of the ICDs for the duration and tachyarrhythmia detection criteria. Antibradycardia pacing was programmed in VVI mode with pacing rate of 40 beats per minute in all VR and DR ICDs, unless the patients needed dual chamber pacing, those with long QT syndrome and hypertrophic obstructive cardiomyopathy.

### Implantation techniques

The implantation routes were either subclavian or Axillary veins percutaneously in electrophysiology laboratory by an electrophysiologist.

### Follow-up protocol

The patients were followed after 1 month postoperatively and every 3 months thereafter and upon receiving high voltage therapy in our outpatient ICD clinic. ICD evaluations during follow-up visits included routine clinically appropriate measurements including interrogation of the device for tachyarrhythmia episodes, evaluation of sensing and pacing thresholds and lead impedance. Floppy diskettes were used to retrieve all episodes of ICD therapy. All the episodes resulted in ICD therapy, studied independently by two electrophysiologists to define the diagnosis. All the episodes were categorized as appropriate or inappropriate. ICD-related complications were documented, as were mortality and cause of death. Chest radiography (CXR) was obtained before hospital discharge and every 6 months to confirm radiographic lead integrity. Clinical evaluation of patients included history and physical examination of the implantation site. Complications were defined as unexpected adverse events that were felt to be related to the implant procedure or to the ICD lead and generator system.

### Statistical analysis

Baseline characteristics and complications were summarized as the mean ± SD for continuous variables and otherwise as percentage. SPSS 13 software (SPSS Inc., Chicago, IL, USA) was used for data storage and analysis. Qualitative characteristics were compared using the Pearson’s chi square and Fischer exact tests. Kaplan-Meyer survival curves were calculated. Log rank statistics were used to compare the distribution of time to complications and mortality between different ICD groups. Two-tailed P < 0.05 were considered significant. Regression analysis was performed to evaluate the effect of variables on mortality and complications.

## Results

### Baseline characteristics

Baseline characteristics of the patients are summarized in Table 1. Table 2 compares the baseline characteristics of the patients in VR and DR ICD groups.

### Mortality

**Figure 1:**
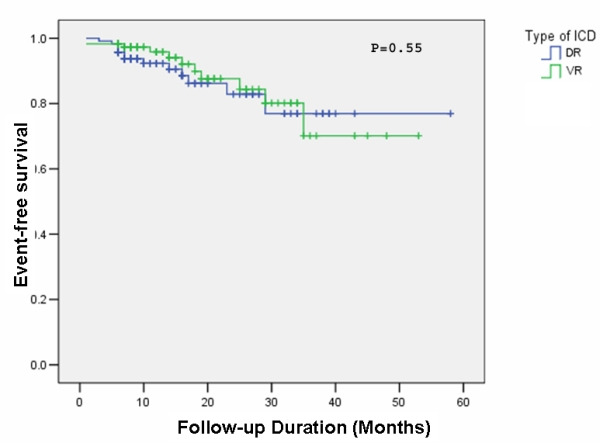
Kaplan-Meier plot of the cumulative survival according to ICD type. Log rank (Mantel-Cox) test was used to generate the P value. ICD-VR= Single-chamber implantable cardioverter-defibrillator; ICD-DR=Dual-chamber implantable cardioverter-defibrillator

Three patients (1.2%) died during the first month after ICD implantation because of multi-organ failure, massive pulmonary emboli and acute coronary syndrome. The 1-year survival rate was 94.9% and 94.1% for VR and DR ICDs respectively. The overall survival rate during follow-up was 90.6% and 88.9% for VR and DR ICDs respectively. There was no significant difference in mortality between different sex groups (P=0.37), different indications for ICD implantation (P=0.43) or between VR and DR ICD groups (P= 0.55) (Figure 1). Univariate regression analysis showed predictors of mortality as NYHA class III or more (P<0.001), age >65 years (P=0.011) and LVEF<30% (P<0.001). The mortality in patients with CAD and DCM were significantly higher than those with other structural heart diseases (P=0.001). During the follow-up, 59 patients (25.2%) had at least one inappropriate ICD therapy. The mortality of this group was significantly higher than those who had not experienced inappropriate therapy (P=0.03).

### Complications

**Figure 2:**
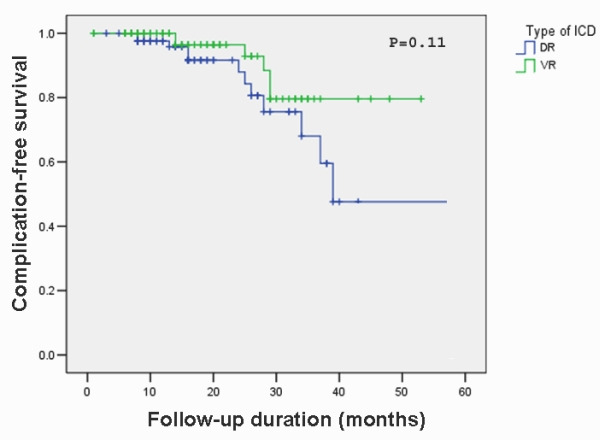
Kaplan-Meier plot of the complication-free survival according to ICD type. Log rank (Mantel-Cox) test was used to generate the P value. ICD-VR= Single-chamber implantable cardioverter-defibrillator; ICD-DR=Dual-chamber implantable cardioverter-defibrillator

Overall, 22 patients (9.4%) suffered from ICD-related complications during follow-up period. There was no significant difference in overall complication rate between VR and DR ICD groups in the follow-up period (P= 0.11) (Figure 2). The risk of complications was not associated with any patient-specific factor including age (P=0.32) and gender (P=0.19) (Table 1). The risk of complications did not have any statistically significant difference in secondary versus primary prevention groups (P=0.06). The complications were not associated with the severity of left ventricular systolic dysfunction (P=0.16). Also, no statistically significant difference was observed between different NYHA classes (P=0.45) and underlying heart diseases (P=0.06). During the follow-up, 59 patients (25.2%) had at least one inappropriate ICD therapy. The risk of complications did not have any statistically significant difference in this group with those who had not experienced inappropriate therapy (P=0.16). The comparison of complications in different models of implanted leads was not possible due to variety of the models and small numbers of each model.

### Implant-related complications

Implant-related complications occurred in 13 patients (5.5%). Hematoma or bleeding was the most frequent complication in this group, found in 7 patients (3%). Pneumothorax and hemothorax were found in 3(1.3%) and 2(0.9%) patients, all of them managed with chest tube insertion. No significant pocket infection was found but only one patient suffered from stitch abscess, treated conservatively with antibiotics.

### Lead-related complications

Lead-related complications occurred in 9 patients (3.8%). Lead dislodgement was the most frequent lead-related complication, found in 5 patients (2.1%). Dislodgement of ventricular and atrial leads was seen in 3 and 2 patients respectively. Lead fracture was found in 2 patients (0.9%). Exit block and insulation break was rare, each of them occurred only in 1 patient (0.4%). 4 of 5 lead dislodgements and all of hematomas occurred in the 2 month of implantation. No cardiac perforation was found. The frequency of lead-related complications was significantly higher in dual chamber ICDs in comparison with single chamber ICDs (P=0.02).

## Discussion

This prospective study characterized the incidence of lead and implant-related complications and mortality of ICD implantations in our center. The baseline demographic data of the patients in this study were comparable to large studies (Table 1) [3-8]. With the downsizing of ICD pulse generators, pectoral implantation has become standard for ICD placement [9]. Older generation pacemakers of comparable size to ICD pulse generators had a significant risk of erosion and other pocket complications [10]. During this study, 234 consecutive ICD implantations over a 33 ±23 months follow-up showed low (9.4%) incidence of complications. The most common implant and lead related complications were hematoma and lead dislodgement which was found in 3% and 2.1% of patients. These findings are consistent with or even rarer than prior report [5-18]. In the majority of previous studies the ICD-related complications did not differ with increasing age which was consistent with this study [15-18]. The infection rate in this case series was very low (0.4%). In large studies, infection rates of 1.3-2.7% were reported. Because most of the infections that were seen in previous reports occurred with abdominal pocket, it seems reasonable to expect a lower infection rate with current generation of ICDs implanted transvenously in the pectoral region [7,20-22]. Intensive use of prophylactic antibiotics before and 48 hours after the procedure and avoidance of cauterization for hemostasis may help us reduce the infection rate.

Some authors encourage the usage of cephalic vein cut downs as the preferred approach for ICD lead venous approach for prevention of vascular complications of blind, percutaneous subclavian venipuncture such as pneumothorax and hemothorax [23,24]. The time course of specific complications reinforces the need for continued lead surveillance in ICD patients. Although bleeding, pneumothorax, lead dislodgement and infection tends to occur soon after implant, lead-related problems such as lead fracture and insulation breaks tend to occur throughout follow-up [7-19,25,26].

## Limitations

The present study must be interpreted in light of certain methodological limitations. The ICDs were implanted with multiple operators of variable training and experience. Therefore, the effects of differences in experience or surgical technique on complication rates were not controlled. However, the inclusion of the large number of cases and relatively long follow-up periods makes it more likely that these results are reliable. The second limitation was lack of cooperation of some patients for regular follow-up and routine CXRs. Therefore, the incidence of lead fractures and insulation breaks may have been underestimated. Also, small hematomas usually managed by sand bag and under- reported.

## Conclusion

This study showed that the pectoral implantation of ICDs by electrophysiologists in our center has a low rate of complications, comparable with most of the qualified centers. Since most of implant-related and some of lead-related complications occur soon after the implant, close early monitoring of patients during first 2 month after implantation is recommended. The continued occurence of lead fractures and the need for premature system revision over time supports the practice of close, routine ICD surveillance. Vascular complications of blind percutaneous subclavian venipuncture could be avoided if cephalic vein cut down is used for ICD lead venous access.

## Figures and Tables

**Table 1:**
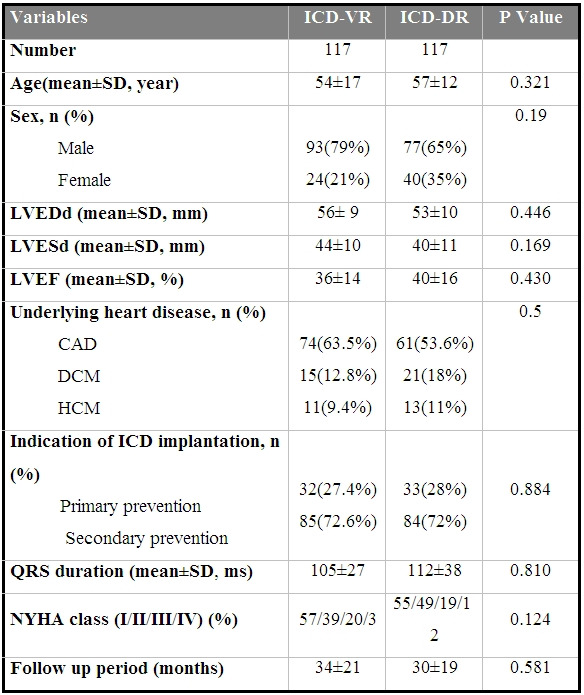
Baseline characteristics of patients

CAD= Coronary artery disease, DCM= Dilated cardiomyopathy, HCM= Hypertrophic cardiomyopathy, NYHA= New York Heart Association

**Table 2:**
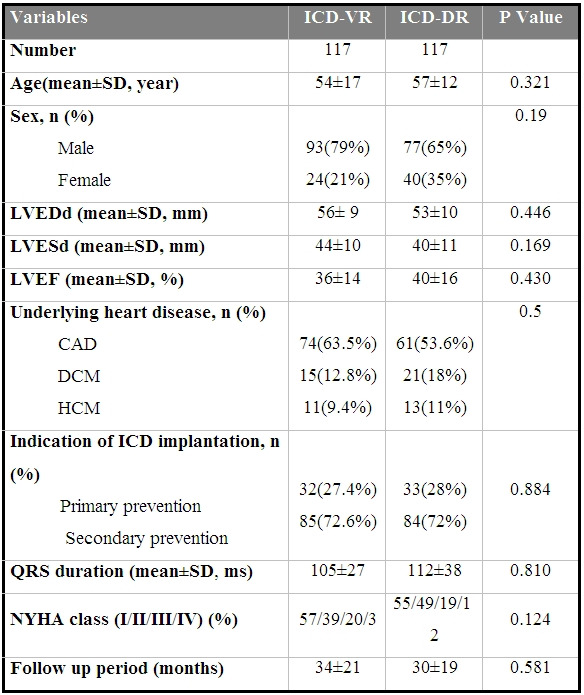
Comparison of baseline characteristics between single and dual chamber devices

ICD-VR= Single chamber implantable cardioverter-defibrillator, ICD-DR=Dual chamber implantable cardioverter-defibrillator, LVEDd= Left ventricular end diastolic diameter, LVESd= Left ventricular end systolic diameter, LVEF= Left ventricular ejection fraction, NYHA= New York Heart Association, CAD= Coronary artery disease, DCM= Dilated cardiomyopathy, HCM= Hypertrophic cardiomyopathy
